# Supplementary material for: Foregut organ progenitors and their niche display distinct viscoelastic properties in vivo during early morphogenesis stages
Source: Commun Biol. 2022 Apr 29;5:402. doi: 10.1038/s42003-022-03349-1 (PMC9054744; doi:10.1038/s42003-022-03349-1)
Supplement: Supplementary file 2 — Supplementary Information [file 42003_2022_3349_MOESM2_ESM.pdf]

SUPPLEMENTARY INFORMATION

Foregut organ progenitors and their niche display distinct viscoelastic properties in vivo during early morphogenesis stages

Dzementsei, Barooji, et al.

SUPPLEMENTARY METHODS

Data quantification and statistical analysis

Power spectral analysis and calculation of the complex shear modulus

The positional power spectrum, $P_x(f)$, of the thermal fluctuations of a tracer inside a viscoelastic environment scale with frequency, *f*. This scaling that is characterized by a scaling exponent α: $P_x(f) \equiv \langle|\tilde{x}(f)|^2\rangle \propto f^{-(1+\alpha)}$ (where, trivially, α= 1 for a tracer in a viscoelastic environment as water)[1]. In the analyses, all power spectra were calculated by custom written MATLAB programs. An average of 3 measurements in *x* and *y* directions was used to extract the value of *α* for each trapped nanoparticle in a single cell. The average of α–values obtained for a specific cell type was then used to infer the viscoelastic properties of the corresponding tissue. Statistical significance of observed differences in viscoelasticity between tissue pairs was determined using a two-tailed equal variance Student's t-test. The significance level was set at 5%. The number of embryos (N) and sample size (n) are indicated in the legend to Figure 5.

The sample size was not computed and decided at the stage of the study design, as the levels of variation and the difference between the samples were hard to predict. However, based on previous publications that used other techniques to measure cell viscoelasticity, we estimated N≥5, n≥40 for unpaired samples (different cell populations) and n≥15 for paired samples (drug treatment) would be reasonable numbers for sample sizes.

To calculate the complex shear modulus, $G(f)$, we followed the procedures outlined in Refs.[2,3]. First, we note that the Fourier transform of the position of a bead in a viscoelastic medium, $x(f)$, is linearly related to the Fourier transform of the thermal force, $F(f)$ [4]:

$$x(f) = \gamma(f)F(f)\ . \qquad (1)$$

Here, $\gamma(f) = \gamma'(f) + i\gamma''(f)$ is the complex response function from which the complex shear modulus, $G(f)$, can be found through the Stokes–Einstein relation:

$$G(f) = G'(f) + iG''(f) = \frac{1}{6\pi r\gamma(f)}, \qquad (2)$$

where r is the radius of the bead, $G'(f)$ denotes the storage modulus, and $G''(f)$ denotes the loss modulus. For a bead in an optical trap, $G'(f)$ is the sum of the storage modulus of the medium and of the optical trap, which exerts a harmonic potential on the particle. If the trap is sufficiently weak, as in current experimental conditions, the elastic contribution from the trap can be ignored.

It has been shown that the response function can be obtain from the mean-squared displacement (M(t)) [2]:

$$2k_B T \text{ɣ}(t) = \frac{d}{dt} M(t)\,. \qquad (3)$$

One can obtain the imaginary and the real part of the response function from the cosine and sine transformations of eq (3):

$$2k_B T \text{ɣ}'(f) = \int_0^\infty cos(2\pi f t) \dot{M}(t)\, dt \qquad (4)$$

and

$$2k_B T \text{ɣ}''(f) = \int_0^\infty sin(2\pi f t) \dot{M}(t)\, dt\,. \quad (5)$$

The methodology is valid in the frequency regime where the tracer particle's fluctuations can be considered to be dominated by thermal fluctuations and where non-equilibrium processes can be neglected.

Using equations (4) and (5), we determined the imaginary and real part of the response function for 400 < *f* < 4000 Hz, a frequency interval fulfilling the above requirement. Via equation (2) the loss and storage moduli were calculated. Both the storage and loss moduli scale with frequency, and this scaling can be described by α , the same scaling exponent describing the scaling of the power spectrum: $G' \sim f^{\alpha}$ and $G'' \sim f^{\alpha}$ [3]. The existence of a finite maximum measurement frequency causes errors to occur in $\gamma(f)$ at sufficiently high frequencies. However, $\gamma(f)$ can be determined with the same bandwidth as the power spectrum[2]. Hence, we used the relation $G'' \sim f^{\alpha}$ to extract α from the shear modulus plot (Figure 2d).

Quantification of microtubule distribution

60 consecutive optical sections obtained every 0.33 µm were used to quantify relative microtubule density in gut and liver for a single embryo. Tissues were manually outlined in Fiji [5] based on *Tg(sox17:GFP)* expression in the endoderm and staining for EfnB1 or Prox1 as a marker for liver progenitors. For higher efficiency, outlining was performed on 1 µm cumulative projections obtained by combining pixel intensity of 3 consecutive 0.33 µm optical sections (in Fiji: Image->Stacks->Z-projection->Sum Slices). Thus, for a single embryo 20 cumulative projections were obtained from 60 consecutive 0.33 µm optical sections corresponding to 20 µm volume. Nuclei were outlined by creating a binary mask based on DAPI intensity with the same threshold for all cumulative projections within a 20 µm volume. Intensity and area were quantified by plotting a histogram of the outlined regions for each cumulative projection (macro

in Fiji: run ("Histogram", "bins=1000 x_min=0 x_max=1000 y_max=Auto")), which returns the number of pixels with a specific value of signal intensity. The bin size was set to 1000, as cumulative projections were generated as a sum of three 8 bit images, and the intensity of a pixel should be ≤ 3x255=765. The area was calculated as a sum of all pixels within the outlined region, while the total signal intensity was obtained as a sum of signal intensity values multiplied by the corresponding pixels number. The total signal intensity and area within a 20 µm volume for gut and liver, as well as for the corresponding nuclei, were quantified as sums of individual cumulative projections. The average signal intensity represents the total signal intensity divided by the area. "Tubulin normalized to DAPI" in Figure 6 represents the average tubulin intensity divided by the average DAPI intensity. Tubulin intensity per cell was calculated as the average tubulin intensity divided by number of nuclei manually calculated within the 20 µm volume. The relative cell area represents the area calculated for gut or liver divided by the number of nuclei within the corresponding tissue. The ratio between gut and liver was calculated for each 20 µm volume individually, and average values represent 3 volumes obtained from different embryos.

Resources and vendors:

REAGENT or RESOURCE	SOURCE	IDENTIFIER
Antibodies		
Polyclonal rabbit anti-EphrinB1	Cayuso et al.,2016	
Polyclonal rabbit anti-Prox1	AngioBio	Catalog #: 11-002
Monoclonal mouse anti-β-tubulin	DSHB; [6]	E7
goat anti-mouse Cy3	Jackson ImmunoResearch Laboratories	115-166-146
goat anti-rabbit Alexa647	Jackson ImmunoResearch Laboratories	111-606-003
Chemicals, Peptides, and Recombinant Proteins		
1-Phenyl-2-thiourea (PTU)	Sigma-Aldrich	P7629; CAS: 103-85-5
Ethyl 3-aminobenzoate methanesulfonate (Tricaine)	Sigma-Aldrich	A5040; CAS: 886-86-2
Instant Ocean sea salt	Aquarium Systems	
SPHERO Fluorescent Particles, Light Yellow, 0.4-0.6 µm	Spherotech	FP-0545-2
NuSieve GTG Agarose, low melting temperature	Lonza	Catalog #: 50085
Dow Corning High Vacuum Grease	Højstrup Industrilim	DC5940-0050
Phalloidin–Atto 633	Sigma-Aldrich	68825

Ultra Pure Agarose, for embedding	Invitrogen	16500-500
PBS Tablets	Gibco	18912-014
VECTASHIELD Antifade Mounting Medium	Vector Laboratories	H-1000
Experimental Models: Organisms/Strains		
Tg(-0.5 sox17:GFP)^{zf99}	Mizoguchi et al., 2008	
Recombinant DNA		
Tol2-Ubi-mKate-CAAX	Courtesy of Sara Caviglia and E.A.O	
Software and Algorithms		
Bitplane IMARIS	Bitplane	http://www.bitplane.com/imarist
Labview	National Instruments	http://www.ni.com/labview/release-archive/2010/
LAS AF Lite	Leica	https://www.leica-microsystems.com
Matlab 2016b	MATLAB	https://www.mathworks.com
Fiji	[5]	http://imagej.net/Fiji
Other		
Nd:YVO4 (λ=1064 nm laser)	Spectra Physics	https://www.spectra-physics.com/
TCS SP5 microscope used for live imaging during optical trapping		https://www.leica-microsystems.com
TCS SP8 microscope used for fixed tissue imaging	Leica microsystems	https://www.leica-microsystems.com
Piezoelectric stage	Mad City Labs	http://www.madcitylabs.com
Photodiode	Hamamatsu	S5981
The Leica VT1000 S microtome	Leica Biosystems	https://www.leicabiosystems.com
Zeiss LSM 880 with Airyscan for high resolution fixed tissue imaging	ZEISS	https://www.zeiss.com

SUPPLEMENTARY FIGURES

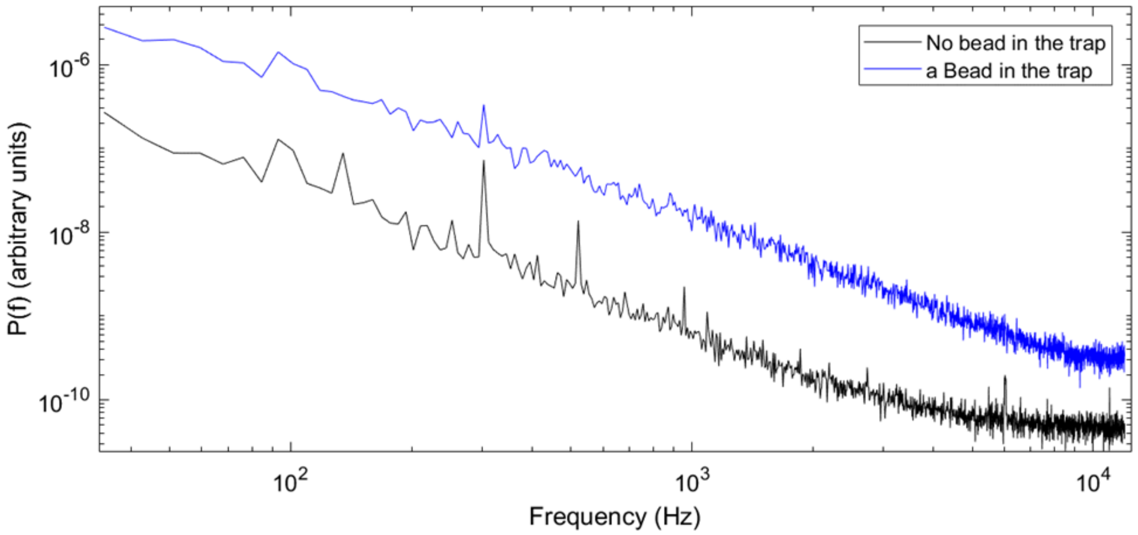

Supplementary Figure 1: Power spectra from measurements inside living zebrafish with (blue) or without (black) a trapped nanoparticle. Due to the focusing effect of the trapped nanoparticle, the amplitude of the power spectrum increases if a nanoparticle is located in the center of the optical trap. The increase in the power spectrum corresponds to an increased signal to noise ratio. Supporting this, noise peaks, e.g., as visible at 300 Hz, are less pronounced upon trapping of a bead.

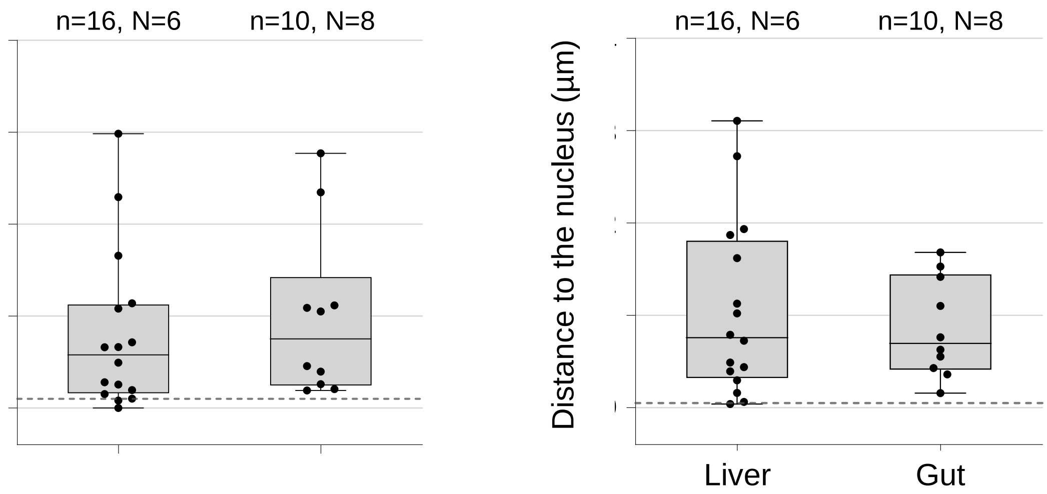

Supplementary Figure 2: Location of tracer particles with respect to the nucleus or actin cortex. High resolution images (see Supplementary Figure 5) were used to measure the distance from the tracer particles to the actin cortex (a) or to the nucleus (b) for particles located in liver or gut progenitors, respectively. n = number of analyzed particles, N = number of embryos. Most investigated particles, regardless of the cell type, are further away from the nucleus and the periphery of the cell (actin cortex) than the amplitude of their thermal motion (~50 nm; dashed line in both panels).

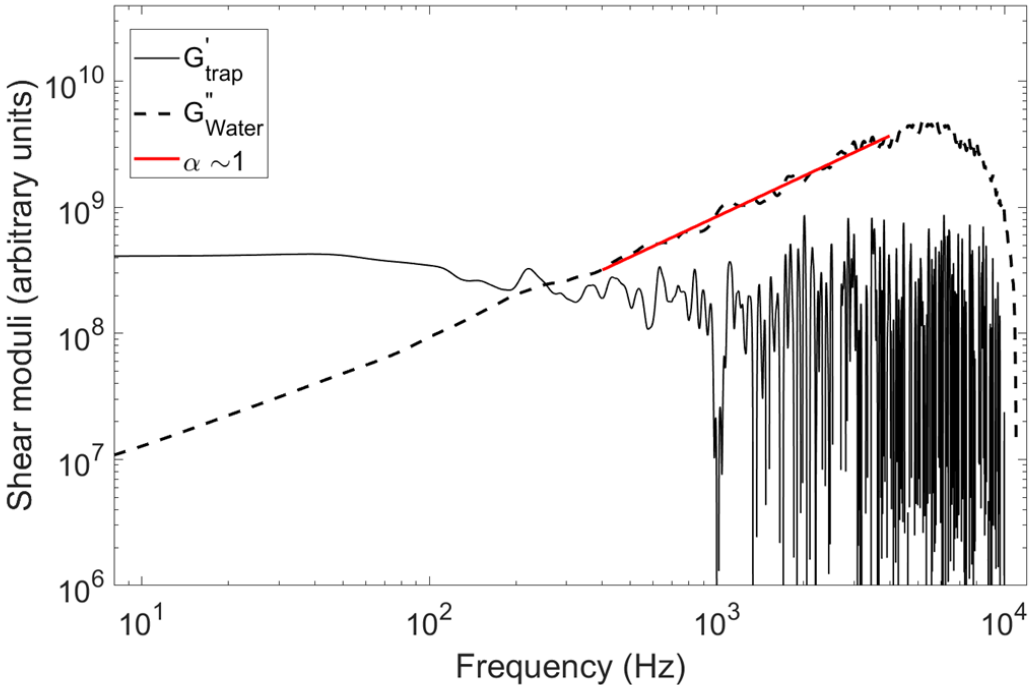

Supplementary Figure 3: Complex shear modulus measured by optical trapping of a bead in water. The harmonic potential of the optical trap gives rise to a storage modulus, *G'* (solid black line), that is frequency independent. As expected for water, a purely viscous liquid, the loss modulus, *G''* (dashed black line), is linearly increasing with frequency with an exponent of α=1 (red line).

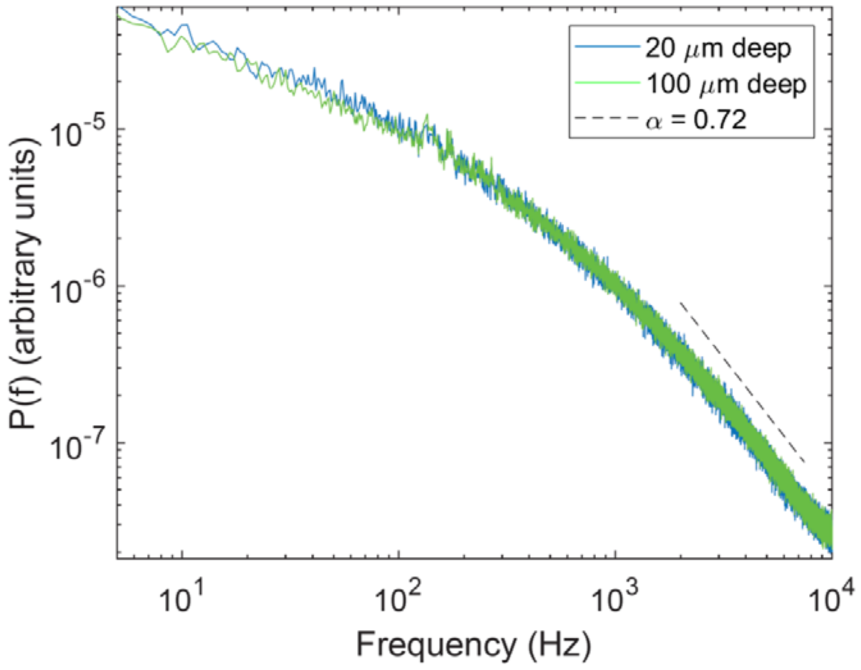

Supplementary Figure 4: Scaling exponents, α, are independent of trapping depth in a uniform viscoelastic medium. Average power spectra for tracer particles trapped at depths of 20 µm (blue) and 100 µm (green) within a uniform viscoelastic polymer gel, experimental details as given in [7]. The slope at relevant frequencies (dashed gray line), providing α, is the same at both depths. The data represent an average of 10 experiments at each depth.

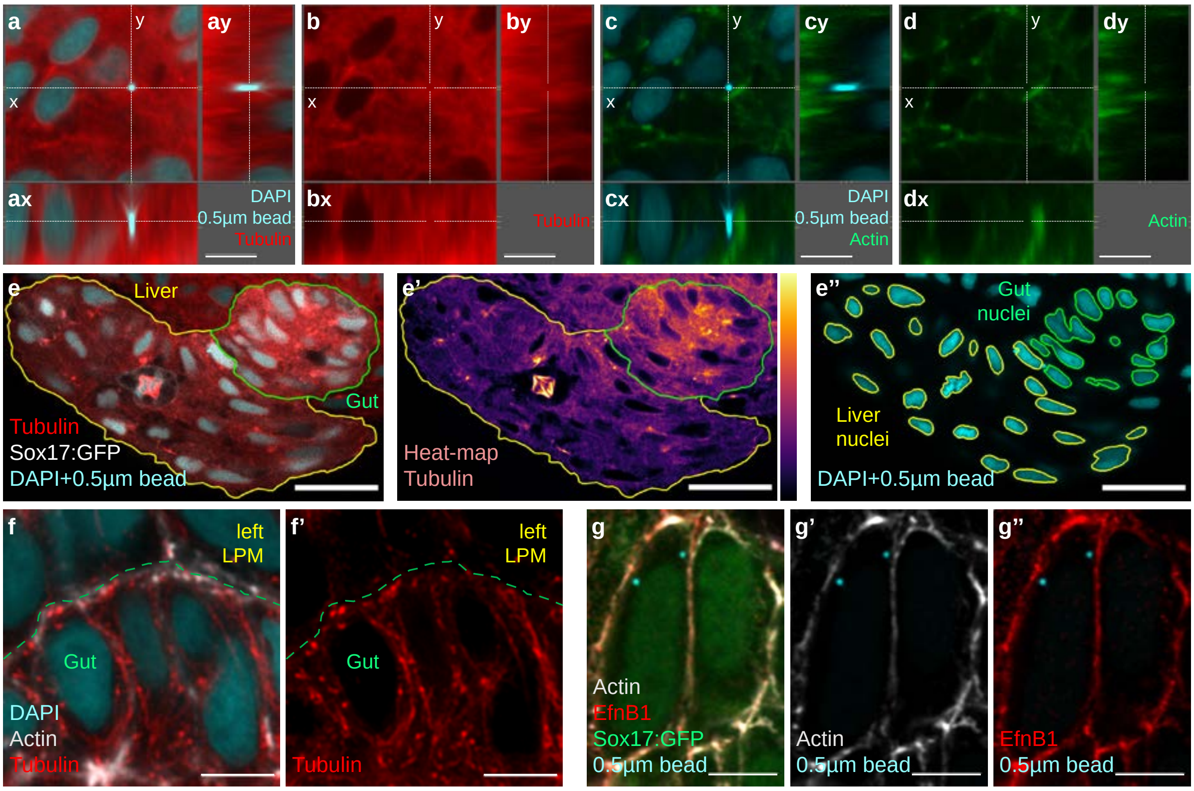

Supplementary Figure 5: Analysis of cellular composition by confocal microscopy. (a-d) Orthogonal projections corresponding to the confocal image shown in Figure 4b. A microinjected nanoparticle (cyan) is surrounded by microtubules (a-b), but not actin filaments (c-d). Scale bar: 5 µm. (e-e'') Representative 1 µm summed projection used to quantify tubulin intensity as shown in Figure 4d. (e) Gut (green line) and liver (yellow line) were manually outlined for each 1 µm summed projection within 20 µm volumes to quantify corresponding tubulin intensity and tissue area. (e') Signal intensity heat-map demonstrates tubulin enrichment in the gut. (e'') Nuclei were outlined by creating a binary mask based on DAPI staining. Nuclear DAPI intensity (as reported in Figure 4d) for both gut and liver was quantified in the outlined nuclei of gut (green) and liver (yellow) progenitors. Scale bar: 20 µm. (f, f') Single optical section showing high-resolution views of microtubules obtained with LSM 880 Airyscan. Dashed line marks the border between gut progenitors and left LPM. Scale bar: 5 µm. (g-g'') Nanoparticles (blue in g) distribute in the cytoplasm between cortical actin and the nucleus as analysed in Supplementary Figure 2; single optical section showing cortical actin filaments (grey) close to the plasma membrane marked by EfnB1 (red) in liver progenitors obtained with LSM 880 Airyscan. Nanoparticles (blue) are localized in the cytoplasm. Scale bar: 5 µm.

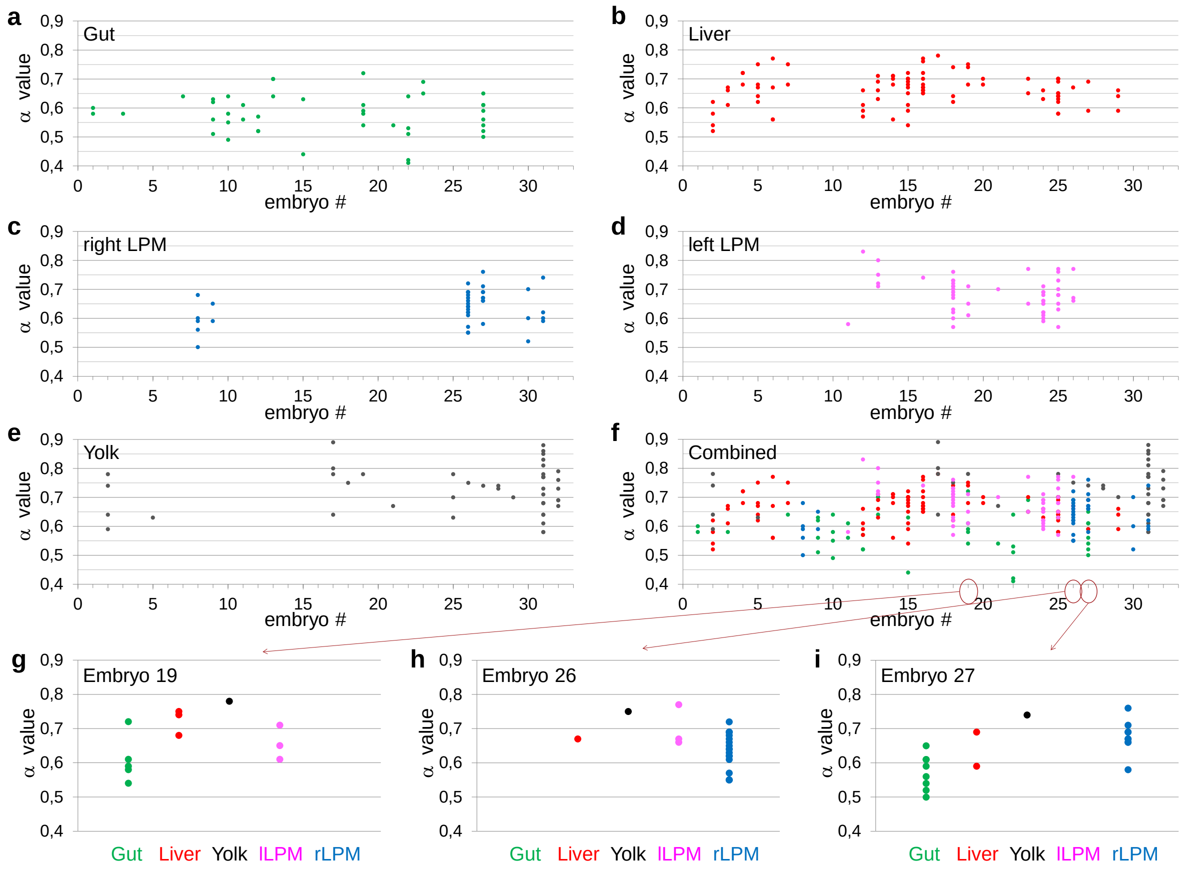

Supplementary Figure 6: Relative distribution of α values in different cell populations is consistent between embryos. Each dot corresponds to the α value measured for a single bead in gut (a, green) or liver (b, red) progenitors, right LPM (c, blue), left LPM (d, magenta), or the yolk (e, black) in one of 32 analysed embryos. (f) Combined distribution of α values of all tissues. (g-i) Relative distribution of α values in three representative individual embryos, grouped by tissue type.

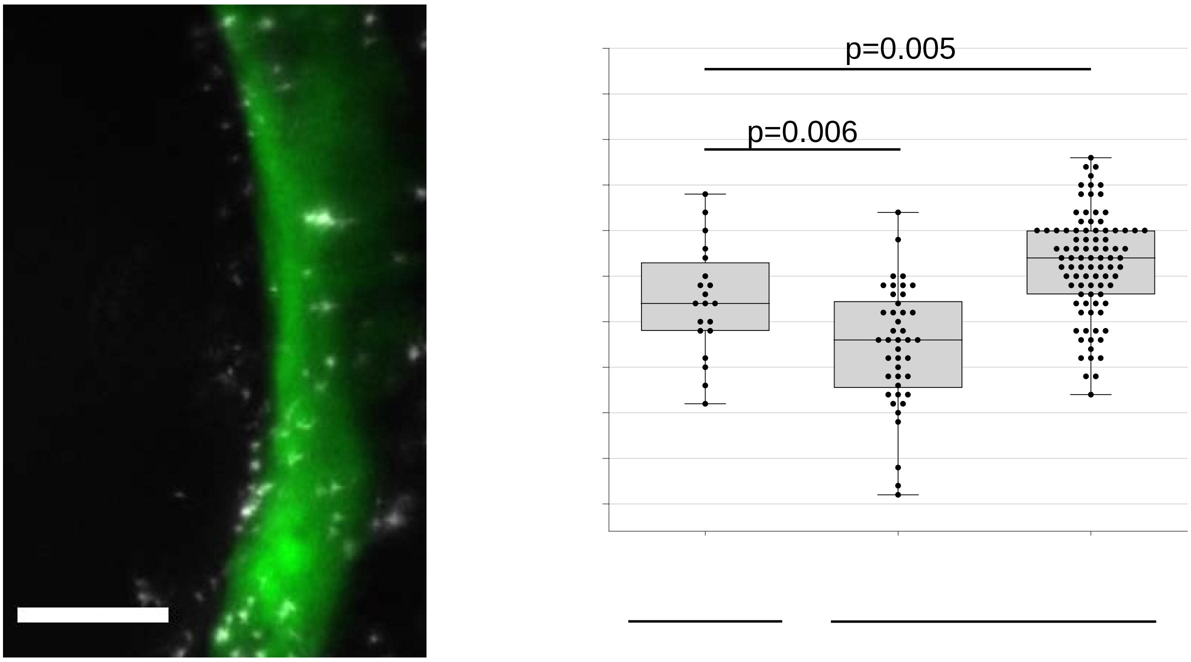

Supplementary Figure 7: Viscoelastic properties of the endoderm at 21-23 hpf. (a) Maximum intensity projection of a confocal z-stack showing the distribution of nanoparticles (white) in the foregut region at 22hpf, *sox17:GFP* marks the endoderm (green); scale bar =50 µm. (b) Comparison of α-values for the endoderm at 21-23 hpf with liver and gut progenitors at 28-30 hpf; n = number of analyzed particles, p-values were calculated using a two-tailed equal variance Student's t-test.

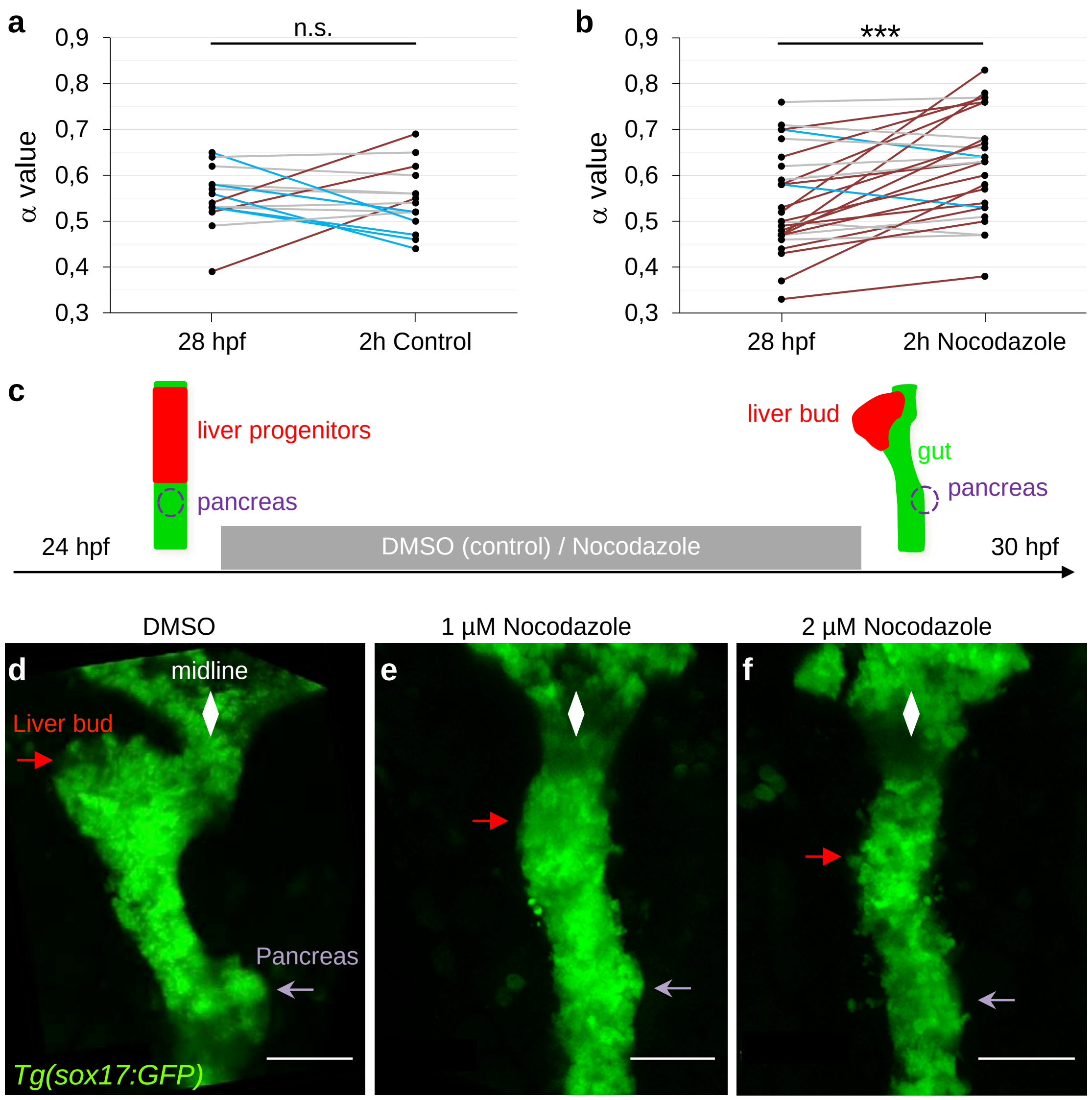

Supplementary Figure 8: Disrupting microtubule polymerisation alters cellular viscoelasticity and foregut morphogenesis. (a-b) α-values determined by sequentially measuring beads in foregut endoderm at 28 and 30 hpf, before and after 2 hours of 2µM Nocodazole treatment (b, N=6, n=26) compared to DMSO controls (a, N=5, n=15). α-value changes: ≥ 0.05 (orange line); -0.05 < difference < 0.05 (grey lines); ≤ -0.05 (blue lines). N=number of embryos, n= number of measured nanoparticles; paired sample 2-tailed t-test: ***p=0.0003, n.s. p=0.8631. (c) Schematic of Nocodazole treatment during gut looping and asymmetric liver bud formation. (d-f) Ventral views of *tg(sox17:GFP)* show how 1 µM and 2 µM Nocodazole treatments impair gut looping and liver bud formation compared to the DMSO control (d); anterior to the top. n=10, N=2.

Supplementary Movie: Optical tweezers trapping a bead in the foregut region of *live* zebrafish. The position of the trap is indicated by a red circle and red points show the locations of beads in the foregut region. The bead-injected embryo was moved very slowly from the left to the right side of the trap, which was kept at a constant position. When a bead came close to the trap, it was pulled into the trap and remained trapped at a constant position for several seconds while the embryo continued to move.

REFERENCES

1 Selhuber-Unkel, C., Yde, P., Berg-Sorensen, K. & Oddershede, L. B. Variety in intracellular diffusion during the cell cycle. *Phys Biol* **6**, doi:Artn 025015 10.1088/1478-3975/6/2/025015 (2009).

2 Nishi, K., Kilfoil, M. L., Schmidt, C. F. & MacKintosh, F. C. A symmetrical method to obtain shear moduli from microrheology. *Soft Matter* **14**, 3716-3723, doi:10.1039/C7SM02499A (2018).

3 Schnurr, B., Gittes, F., MacKintosh, F. C. & Schmidt, C. F. Determining Microscopic Viscoelasticity in Flexible and Semiflexible Polymer Networks from Thermal Fluctuations. *Macromolecules* **30**, 7781-7792, doi:10.1021/ma970555n (1997).

4 Gittes, F., Schnurr, B., Olmsted, P. D., MacKintosh, F. C. & Schmidt, C. F. Microscopic Viscoelasticity: Shear Moduli of Soft Materials Determined from Thermal Fluctuations. *Phys Rev Lett* **79**, 3286-3289, doi:10.1103/PhysRevLett.79.3286 (1997).

5 Schindelin, J. *et al.* Fiji: an open-source platform for biological-image analysis. *Nat Methods* **9**, 676-682, doi:10.1038/nmeth.2019 (2012).

6 Chu, D. T. & Klymkowsky, M. W. The appearance of acetylated alpha-tubulin during early development and cellular differentiation in Xenopus. *Dev Biol* **136**, 104-117 (1989).

7 Borries, M., Barooji, Y. F., Yennek, S., Grapin-Botton, A. & Oddershede, L. B. Quantification of Visco-Elastic Properties of a Matrigel for Organoid Development as a Function of Polymer Concentration. *Frontiers in Physics* **8**, doi:10.3389/fphy.2020.579168 (2020).
