# Supplementary material for: Foregut organ progenitors and their niche display distinct viscoelastic properties in vivo during early morphogenesis stages
Source: Commun Biol. 2022 Apr 29;5:402. doi: 10.1038/s42003-022-03349-1 (PMC9054744; doi:10.1038/s42003-022-03349-1)
Supplement: Supplementary file 3 — Description of Additional Supplementary Files [file 42003_2022_3349_MOESM3_ESM.pdf]

Description of Additional Supplementary Files

File name: Supplementary Data
Description: Source data underlying the graphs presented in this study.

File name: Supplementary Movie
Description: Optical tweezers trapping a bead in the foregut region of live zebrafish.

The position of the trap is indicated by a red circle and red points show the locations of beads in the foregut region. The bead-injected embryo was moved very slowly from the left to the right side of the trap, which was kept at a constant position. When a bead came close to the trap, it was pulled into the trap and remained trapped at a constant position for several seconds while the embryo continued to move.
