# Supplementary material for: Foregut organ progenitors and their niche display distinct viscoelastic properties in vivo during early morphogenesis stages
Source: Commun Biol. 2022 Apr 29;5:402. doi: 10.1038/s42003-022-03349-1 (PMC9054744; doi:10.1038/s42003-022-03349-1)
Supplement: Supplementary file 6 — Reporting summary [file 42003_2022_3349_MOESM6_ESM.pdf]

nature portfolio

Corresponding author(s): DBPR COMMSBIO-21-1395

Last updated by author(s): Apr 3, 2022

Reporting Summary

Nature Portfolio wishes to improve the reproducibility of the work that we publish. This form provides structure for consistency and transparency in reporting. For further information on Nature Portfolio policies, see our Editorial Policies and the Editorial Policy Checklist.

Statistics

For all statistical analyses, confirm that the following items are present in the figure legend, table legend, main text, or Methods section.

n/a	Confirmed	
☐	☒	The exact sample size (*n*) for each experimental group/condition, given as a discrete number and unit of measurement
☐	☒	A statement on whether measurements were taken from distinct samples or whether the same sample was measured repeatedly
☐	☒	The statistical test(s) used AND whether they are one- or two-sided<br>*Only common tests should be described solely by name; describe more complex techniques in the Methods section.*
☐	☒	A description of all covariates tested
☐	☒	A description of any assumptions or corrections, such as tests of normality and adjustment for multiple comparisons
☐	☒	A full description of the statistical parameters including central tendency (e.g. means) or other basic estimates (e.g. regression coefficient) AND variation (e.g. standard deviation) or associated estimates of uncertainty (e.g. confidence intervals)
☐	☒	For null hypothesis testing, the test statistic (e.g. F, t, r) with confidence intervals, effect sizes, degrees of freedom and P value noted<br>*Give P values as exact values whenever suitable.*
☒	☐	For Bayesian analysis, information on the choice of priors and Markov chain Monte Carlo settings
☒	☐	For hierarchical and complex designs, identification of the appropriate level for tests and full reporting of outcomes
☒	☐	Estimates of effect sizes (e.g. Cohen's d, Pearson's r), indicating how they were calculated

Our web collection on statistics for biologists contains articles on many of the points above.

Software and code

Policy information about availability of computer code

Data collection	Labview and standard Leica software, LAS AF Lite, Zen (black) for Zeiss LSM780 and LSM 880 microscopes, was used to collect data
Data analysis	Data was analyzed by standard Matlab software, Microsoft Excel, Fiji (Schindelin, J. et al. Nat Meth 9, 676–682 (2012), and Bitplane IMARIS

For manuscripts utilizing custom algorithms or software that are central to the research but not yet described in published literature, software must be made available to editors and reviewers. We strongly encourage code deposition in a community repository (e.g. GitHub). See the Nature Portfolio guidelines for submitting code & software for further information.

Data

Policy information about availability of data

All manuscripts must include a data availability statement. This statement should provide the following information, where applicable:

- Accession codes, unique identifiers, or web links for publicly available datasets
- A description of any restrictions on data availability
- For clinical datasets or third party data, please ensure that the statement adheres to our policy

The datasets generated during and/or analyzed during the current study are available from the corresponding authors on reasonable request.

Field-specific reporting

Please select the one below that is the best fit for your research. If you are not sure, read the appropriate sections before making your selection.

☒ Life sciences ☐ Behavioural & social sciences ☐ Ecological, evolutionary & environmental sciences

For a reference copy of the document with all sections, see nature.com/documents/nr-reporting-summary-flat.pdf

Life sciences study design

All studies must disclose on these points even when the disclosure is negative.

Sample size	The exact sample sizes used for each of the different analysis are described in detail in the manuscript.
Data exclusions	No data was excluded.
Replication	Replications were successful.
Randomization	Samples were randomly allocated.
Blinding	Investigators were blinded to group allocations.

Reporting for specific materials, systems and methods

We require information from authors about some types of materials, experimental systems and methods used in many studies. Here, indicate whether each material, system or method listed is relevant to your study. If you are not sure if a list item applies to your research, read the appropriate section before selecting a response.

Materials & experimental systems

n/a	Involved in the study	
☐	☒	Antibodies
☒	☐	Eukaryotic cell lines
☒	☐	Palaeontology and archaeology
☐	☒	Animals and other organisms
☒	☐	Human research participants
☒	☐	Clinical data
☒	☐	Dual use research of concern

Methods

n/a	Involved in the study	
☒	☐	ChIP-seq
☒	☐	Flow cytometry
☒	☐	MRI-based neuroimaging

Antibodies

Antibodies used	mouse α-Prox1 (1:50; Abcam, cat# ab33219, lot#GR296243-3)<br>rabbit α-EphrinB1 (custom-made by Ober group; Cayuso et al. 2016)<br>mouse β-tubulin antibody (1:50; E7, DSHB)
Validation	mouse α-Prox1 (1:50; Abcam), identical staining pattern to rabbit α-Prox1 (1:500; Angiobio)<br>rabbit α-EphrinB1, validation by staining in loss-of-function mutant (Cayuso et al. Dev Cell 2016)<br>mouse β-tubulin antibody (E7, DSHB) (Rhinn et al. Neural Development 2009)

Animals and other organisms

Policy information about studies involving animals; ARRIVE guidelines recommended for reporting animal research

Laboratory animals	Species: zebrafish (Danio rerio) embryos and larvae; adults carriers for breeding<br>Strain: Tg(-0.5 sox17:GFP)zf99 (Mizoguchi et al 2008)
Wild animals	*Provide details on animals observed in or captured in the field; report species, sex and age where possible. Describe how animals were caught and transported and what happened to captive animals after the study (if killed, explain why and describe method; if released, say where and when) OR state that the study did not involve wild animals.*
Field-collected samples	*For laboratory work with field-collected samples, describe all relevant parameters such as housing, maintenance, temperature, photoperiod and end-of-experiment protocol OR state that the study did not involve samples collected from the field.*
Ethics oversight	Zebrafish experiments were performed according to ethical guidelines approved by the Danish Animal Experiments Inspectorate (Dyreforsøgstilsynet).

Note that full information on the approval of the study protocol must also be provided in the manuscript.
